# Long-term complications and outcomes of therapeutic embolization of cerebral arteriovenous malformations: a systematic review

**DOI:** 10.1590/1516-3180.2022.0591.R1.20022024

**Published:** 2024-07-15

**Authors:** Vivianne Beatriz dos Santos Lúcio, Vinício Rufino Queiroz, Cícero José Pacheco Lins, Jussara Almeida de Oliveira Baggio, Carlos Dornels Freire de Souza

**Affiliations:** I Undergraduate Student, Universidade Federal de Alagoas (UFAL), Arapiraca (AL), Brazil.; II Undergraduate Student, Universidade Federal de Alagoas (UFAL), Arapiraca (AL), Brazil.; III Neurosurgery, Ebersh, Hospital Universitário Professor Alberto Antunes (UFAL), Maceió (AL), Brazil.; IV Assistant Professor, Medicine school, Universidade Federal de Alagoas (UFAL), Arapiraca (AL), Brazil.; V Assistant Professor, Collegiate of Medicine, Universidade Federal do Vale do São Francisco (UNIVASP), Petrolina (PE), Brazil.

**Keywords:** Central nervous system vascular malformations., Embolization, therapeutic., Cerebrovascular disorders., Neurosurgery., Brain arteriovenous malformations., Endovascular treatment., Complications., Outcomes., Long term., Vascular disorders.

## Abstract

**BACKGROUND:**

Embolization is a promising treatment strategy for cerebral arteriovenous malformations (AVMs). However, consensus regarding the main complications or long-term outcomes of embolization in AVMs remains lacking.

**OBJECTIVE:**

To characterize the most prevalent complications and long-term outcomes in patients with AVM undergoing therapeutic embolization.

**DESIGN AND SETTING:**

This systematic review was conducted at the Federal University of Alagoas, Arapiraca, Brazil.

**METHODS:**

This systematic review was conducted according to the Preferred Reporting Items for Systematic Reviews and Meta-Analyses criteria. Data were obtained from MEDLINE, PubMed, LILACS, and SciELO databases, which included the epidemiological profile of the population, characteristics of the proposed therapy, complications (hemorrhagic events and neurological deficits), and long-term outcomes (modified Rankin scale pre- and post-treatment, AVM recanalization, complete obliteration, and deaths).

**RESULTS:**

Overall, the analysis included 34 articles (2,799 patients). Grade III Spetzler–Martin AVMs were observed in 34.2% of cases. Notably, 39.3% of patients underwent embolization combined with radiosurgery. The most frequently reported long-term complication was hemorrhage, which occurred in 8.7% of patients at a mean follow-up period of 58.6 months. Further, 6.3% of patients exhibited neurological deficits after an average of 34.7 months. Complete obliteration was achieved in 51.4% of the cases after a mean period of 36 months. Recanalization of AVMs was observed in 3.5% of patients. Long-term death occurred in 4.0% of patients.

**CONCLUSION:**

Embolization of AVMs is an increasingly safe strategy with low long-term complications and satisfactory outcomes, especially in patients who have undergone combination therapies.

**SYSTEMATIC REVIEW REGISTRATION:**

https://www.crd.york.ac.uk/prospero/ Registration number CRD42020204867.

## INTRODUCTION

Cerebral arteriovenous malformations (AVMs) are anomalous communications between cerebral arteries and veins, a complex capillary network, resulting in an increased risk of intraparenchymal hemorrhage.^1^ Other hemodynamic changes include venous hypertension, reversal of venous flow, and hypoperfusion of areas adjacent to the AVM.^2,3^

AVM is a rare condition, with an incidence of approximately one case per year per 100,000 people and 2–4% of the annual risk of hemorrhage, accounting for 5–25% of the mortality rate.^2-4^ Neurological deficits occur in 10–50% of cases following the hemorrhage.^2-4^ Notably, the risk of cerebral bleeding is higher in patients with previous bleeding and AVMs deeply located in the brain.^4^

Four therapeutic strategies are employed for AVMs. In some cases, conservative management can be adopted, with doctors and patients aware of the risk of bleeding and the appearance of other symptoms. The intervention options include microsurgical resection, radiosurgery, and therapeutic embolization, which can be used alone or in combination. Nevertheless, the risks and benefits of each therapeutic alternative must be assessed individually during patient assessment.^5-7^

Embolization stands out among treatment options. Currently, embolization can be used as a single therapeutic modality or as an adjunctive therapy to microsurgery or radiosurgery to block arterial blood flow in AVMs, thus reducing their size and high-risk characteristics.^8,9^ However, this type of intervention can cause long-term complications, and outcomes vary among patients. The characterization of complications and outcomes can facilitate the selection of the best therapeutic intervention to achieve higher rates of complete obliteration and cure.

## OBJECTIVE

This systematic review was aimed at analyzing the literature on the most prevalent long-term complications and outcomes in patients with AVMs undergoing therapeutic endovascular embolization.

## METHODS

### Articles search

This systematic review was performed according to the guidelines of the Preferred Reporting Items for Systematic Reviews and Meta-Analyses and was registered in the PROSPERO—International Prospective Register of Systematic Reviews—under the registration number CRD42020204867. Two independent researchers searched for articles published from February to July 2020 in the electronic databases MEDLINE, PubMed, LILACS, and SciELO.

The keywords used for the research were “Intracranial Arteriovenous Malformations” and “Therapeutic Embolization” in conjunction with the Boolean operators AND and OR. Combinations were made using the same keywords, such as “Embolization, Therapeutic/Adverse Effects,” “Embolization, Therapeutic/Epidemiology,” “Embolization, Therapeutic/Rehabilitation,” “Embolization, Therapeutic/Statistics and Numerical Data,” “Intracranial Arteriovenous Malformations/Therapy,” and “Intracranial Arteriovenous Malformations/Surgery.” The following filters were used for the type of study: “Clinical Study,” “Clinical Trial,” “Observational Study,” and “Randomized Controlled Trial.” All keywords and filters were written in English, Spanish, and Portuguese. Notably, we did not identify any previous reviews on this topic.

### Articles selection

Articles with an evaluation of at least one long-term outcome or complication in patients diagnosed with AVMs and treated with therapeutic embolization in isolation or combined with other therapeutic strategies (radiosurgery and/or microsurgery) were included in this review. The exclusion criteria were as follows: 1) studies describing other intracranial AVMs, such as cavernomas, developmental venous anomalies, Galen’s vein malformations, and dural fistulas; 2) studies with no therapeutic embolization; 3) studies with a lack of evidence for long-term follow-up of patients; 4) studies that referred to short-term complications/outcomes (up to 30 days after treatment); 5) technical notes, experience reports, and literature reviews; and 6) publications in languages other than English, Portuguese, and Spanish.

The initial inclusion eligibility of identified studies was assessed by two independent authors based on the titles and abstracts. Subsequently, articles with titles and abstracts meeting the inclusion criteria were selected for further review. Thereafter, the same two authors read the full texts and selected eligible articles based on the inclusion and exclusion criteria. Any disagreements between the two authors were analyzed and resolved by a third or fourth author. The reference lists of the selected articles were revised to identify potentially relevant articles that were lost during online searches.

### Data extraction

The data were initially extracted and typed on a spreadsheet by one author and confirmed by another author to ensure accuracy. The extracted data included: 1) population profile of each study (number of participants, age, sex, initial presentation, presence of aneurysms, and Spetzler–Martin scale); 2) characterization of the proposed therapy (type of embolizing material, number of embolizations, and approach used for embolization); 3) follow-up time and number of participants lost; 4) long-term complications and outcomes (intracranial hemorrhages, neurological deficits, recanalization of AVMs, effects of radiosurgery, complete obliteration, death, and other outcomes); 5) modified Rankin scale (mRs) score pre- and post-treatment.

### Data synthesis and evaluation of study quality

Outcomes that occurred within and after 30 days following the therapeutic intervention were considered short- and long-term outcomes and complications, respectively. Descriptive statistics were employed to calculate the rates and averages for each study. Quality analysis of the selected studies was performed using the Newcastle–Ottawa Scale.^10^ This scale has three domains: selection (maximum of four stars), comparability (maximum of two stars), and results (maximum of three stars). A study is considered to have strong evidence if it receives three or four, one or two, and two or three stars in the selection, comparability, and results domains, respectively. Moderate evidence is considered if a study receives two, one or two, and two or three stars in the selection, comparability, and results domains, respectively. Limited evidence is considered if a study receives zero or one, zero, and zero or one stars in the selection, comparability, and results domains, respectively.

## RESULTS

**Figure 1:**
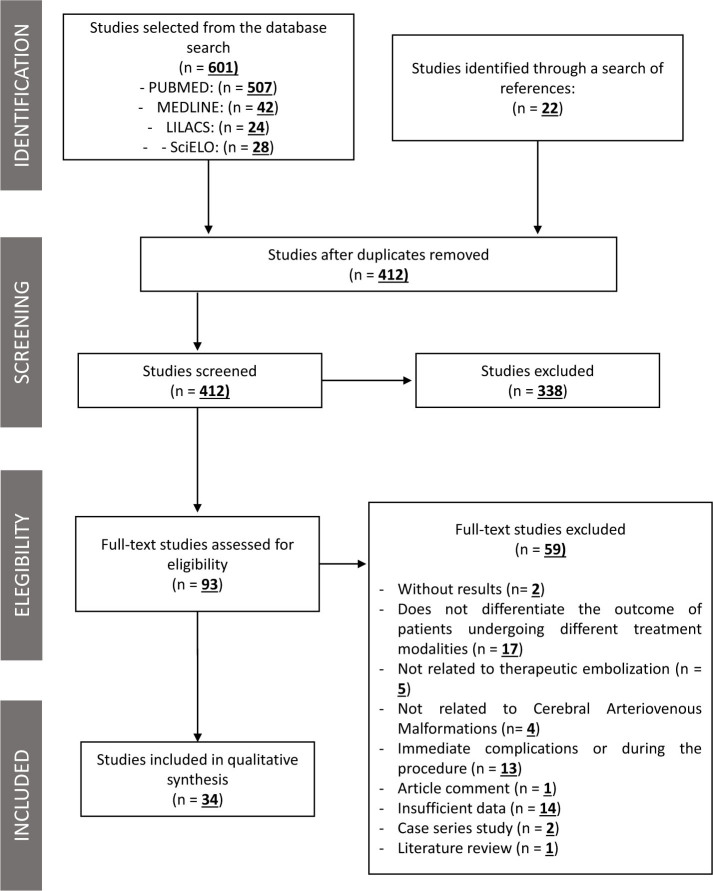
Flowchart from studies selection.

**Figure 2:**
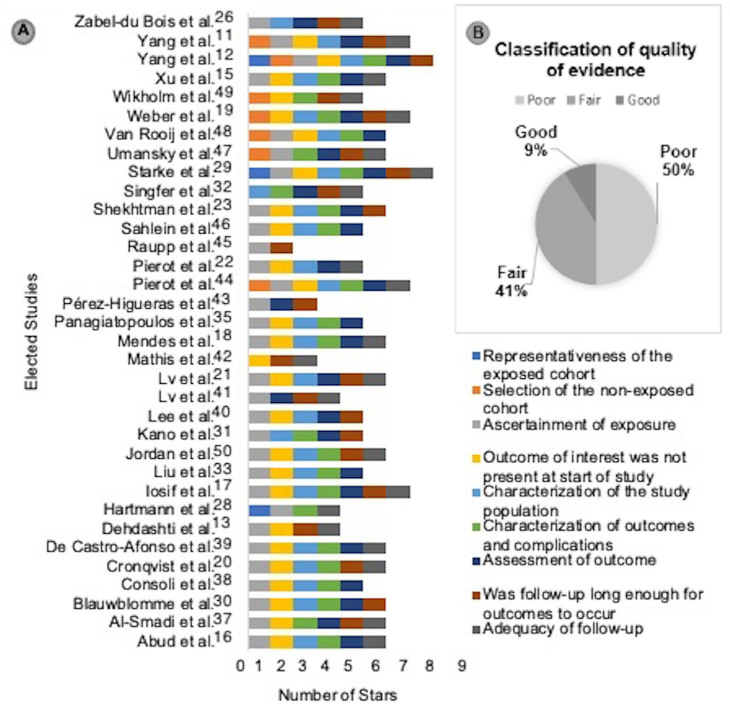
Assessment of methodological quality of studies using the Newcastle Ottawa Scale.

We obtained 601 studies from the database searches (PubMed, 507; MEDLINE, 42; LILACS, 24; SciELO, 28). Another 22 articles were selected from the reference lists of the pre-selected studies. A total of 412 articles remained after eliminating the duplicates. Further, 338 articles were excluded based on titles and abstracts, leaving 93 articles for full-text reading. Of these, 58 articles that did not meet the inclusion criteria were excluded. Finally, 34 articles were included in this systematic review (5 case-control studies, 15 retrospective cohorts, and 14 prospective cohorts) (**Figure 1**). The quality analysis of each study included in this review is summarized in **Figure 2**.

The data of 2,799 patients with AVMs treated using embolization therapy alone or in combination with other treatments were assessed in this review. The mean age of patients was 32.3 years (range 9.7–44.2 years), and 52.2% were male and 47.8% were female (**Table 1**). The most prevalent clinical presentation was hemorrhage (42.6%), followed by headaches (32.4%), seizures (30.6%), and neurological deficits (12%). Notably, 116 (4.4%) asymptomatic patients at the time of admission were also identified from the selected studies. Patients also presented with more than one clinical presentation on admission. Additionally, other symptoms such as visual disturbances, weakness, speech disorders, and imbalance are also possible, as reported by Yang et al.^11^ and Yang et al.^12^

**Table 1:** Characteristics of studies included in the systematic review and patients with cerebral arteriovenous malformations

Reference	Type of study	Characteristics of the patients			
		Number	Age (Years, mean)	Sex	
				M	F
Yang et al.^11^	Cohort (retrospective)	31	26.4	15	16
Yang et al.^12^	Cohort (retrospective)	420	35.0	180	240
Dehdashti et al.^13^	Case-control	135	36.0	70	65
Singfer et al.^14^	Cohort (retrospective)	61	38.0	32	29
Xu et al.^15^	Cohort (prospective)	86	30.3	51	35
Abud et al.^16^	Cohort (retrospective)	17	32.7	8	9
Iosif et al.^17^	Cohort (prospective)	73	40.5	45	28
Mendes et al.^18^	Cohort (prospective)	7	13.5	3	4
Weber et al.^19^	Cohort (retrospective)	47	36.0	31	16
Cronqvist et al.^20^	Cohort (prospective)	21	40.7	15	6
Lv et al.^21^	Cohort (retrospective)	144	27.9	92	52
Pierot et al.^22^	Cohort (retrospective)	20	33.2	9	11
Shekhtman et al.^23^	Case-control	40	32.1	24	16
Zabel-du Bois et al.^26^	Cohort (retrospective)	50	37.2	27	23
Hartmann et al.^28^	Cohort (prospective)	233	36.0	109	124
Starke et al.^29^	Cohort (prospective)	202	35.0	84	118
Blauwblomme et al.^30^	Cohort (prospective)	106	9.7	62	44
Kano et al.^31^	Case-control	120	33.0	61	59
Liu et al.^33^	Cohort (prospective)	31	32.6	19	12
Panagiatopoulos et al.^35^	Cohort (retrospective)	82	44.2	41	41
Al-Smadi et al.^37^	Cohort (retrospective)	34	10.6	22	12
Consoli et al.^38^	Cohort (prospective)	84	38.2	52	32
de Castro-Afonso et al.^39^	Cohort (retrospective)	23	11.7	11	12
Lee et al.^40^	Case-control	25	42.0	15	10
Lv et al.^41^	Cohort (retrospective)	30	31.0	22	8
Mathis et al.^42^	Cohort (prospective)	24	N/S	N/S	N/S
Pérez-Higueras et al.^43^	Cohort (prospective)	45	35.0	22	23
Pierot et al.^44^	Cohort (prospective)	117	42.6	72	45
Raupp et al.^45^	Cohort (prospective)	104	30.0	59	45
Sahlein et al.^46^	Cohort (retrospective)	130	34.2	64	66
Umansky et al.^47^	Cohort (retrospective)	12	N/S	N/S	N/S
van Rooij et al.^48^	Cohort (retrospective)	24	41.0	17	7
Wikholm et al.^49^	Cohort (prospective)	150	35.5	67	83
Jordan et al.^50^	Cohort (prospective)	71	N/S	41	30
**Total**		2,799	32.3	1,442 (52.2%)	1,321 (47.8%)

N/S = not specified; M = Male; F = Female.

Of the selected articles, 13 provided information about the mRs score before treatment. Of the 1002 patients, 90.2% (n = 908) had a mRs score of 0–2. Further, 25 studies classified AVMs according to the Spetzler–Martin scale, with a total of 2,130 cases. Of these, 16.1% (n = 343), 30.8% (n = 656), 34.2% (n = 728), 15.5% (n = 331), 2.7% (n = 58), and 0.7% (n = 14) were grade I, II, III, IV, V, and VI Spetzler–Martin AVMs, respectively. Additionally, 16 studies (1,731 patients) reported the presence of aneurysms associated with AVMs, with a prevalence rate of 26.2% (n = 454). No information was collected regarding aneurysm characteristics (**Table 2**).

**Table 2:** Clinical and angiographic profiles of patients with cerebral arteriovenous malformations

Variable	Subgroup	Number of studies	Total number of patients	Number of patients per subgroup	%
Initial clinical presentation	Asymptomatic	32	2,609	116	4.4%
	Hemorrhage	32	2,609	1,113	42.6%
	Neurological deficit	32	2,609	313	12.0%
	Headache	32	2,609	845	32.4%
	Seizure	32	2,609	799	30.6%
	Others	2	451	257^*^	57.0%
Modified Rankin scale score (mRs; pre-treatment)	mRs 0–2	13	1,002	908	90.6%
	mRs >2	13	1,002	94	9.4%
Angiographic presentation	Spetzler–Martin Grade I	25	2,130	343	16.1%
	Spetzler–Martin Grade II	25	2,130	656	30.8%
	Spetzler–Martin Grade III	25	2,130	728	34.2%
	Spetzler–Martin Grade IV	25	2,130	331	15.5%
	Spetzler–Martin Grade V	25	2,130	58	2.7%
	Presence of brain aneurysms	16	1,731	454	26.2%
Proposed treatment	Isolated embolization	34	2,589	961	37.1%
	Embolization + Radiosurgery	34	2,589	1017	39.3%
	Embolization + Microsurgery	34	2,589	594	23%
	Embolization + Radiosurgery + Microsurgery	34	2,589	17	0.6%

* Visual disturbances (82 cases), weakness (73 cases), speech disorders (67 cases), and imbalance (35 cases).

A total of 2,589 patients were treated in the reviewed studies. Of these, 37.1% (n = 961) underwent embolization alone, 39.3% (n = 1,017) underwent embolization and radiosurgery, and 23% (n = 594) underwent embolization and microsurgery. Only 17 patients (0.6%) underwent three combined strategies (embolization, radiosurgery, and microsurgery). Data of patients who were treated with the three therapies were presented only by Dehdashti et al.,^13^ Singfer et al.,^14^ Xu et al.,^15^ and Yang et al.^12^(**Table 2**).

The approach used for vascular access in therapeutic embolization was not specified in most studies. Of the studies that specified the approach, Abud et al.^16^ and Iosif et al.^17^ used an arterial approach, whereas Mendes et al.^18^ and Weber et al.^19^ used a combined arterial and venous approach. Twenty studies reported the use of Onyx as an embolic material, and seven studies used it in combination with other materials. Other embolic materials include N-butyl-2-cyanoacrylate, synthetic glue based on cyanoacrylate (Glubran), polyvinyl alcohol, and springs. Dehdashti et al.,^13^ Yang et al.,^11^ and Yang et al.^12^ did not specify the type of material used for embolization. The average number of embolization sessions per patient was approximately two.

Analyzing long-term complications, 12 studies reported bleeding events with a prevalence of 8.7% (112/1,291 patients). The mean follow-up period in these 12 studies was 58.6 months. Cronqvist et al.,^20^ Lv et al.,^21^ and Pierot et al.^22^ reported the variations in long-term complications during the follow-up period (**Table 3**). Fourteen studies reported neurological deficits as a long-term complication, and six did not specify the nature of the deficits. A total of 81 neurological deficits were reported in 1,291 patients (6.3%). The mean follow-up period was 34.7 months. The most common deficits were hemiparesis, speech disorders (aphasia and dysarthria), visual disturbances (hemianopia and diplopia), ataxia, paresthesia, facial paralysis, permanent weakness in the right hand, and unilateral deafness (one case) (**Table 3**).

**Table 3:** Long-term complications and outcomes of included patients in the systematic review

Complications and outcomes	Number of studies	Total number of patients	Patients per complications and outcome	%	Follow-up (months, mean)
Hemorrhagic events	12	1,291	112	8.7%	58.6
Neurological deficits	14	1,291	81^*^	6.3%	34.7
Complete obliteration/cure	23	1,071	552	51.4%	36.0
Recanalization	5	231	8	3.5%	18.1
Death	9	889	36	4%	N/S
Modified Rankin scale (mRs) score 0–2 (post-treatment)	12	636	589	92.6%	N/S
Modified Rankin scale (mRs) score > 2 (post-treatment)	12	636	47	7.4%	N/S
Reference	Other complications and outcomes				
Dehdashti et al.^13^	Total improvement of headache after treatment: C = 28 patients (67%); CT = 8 patients (20%)				
Hartmann et al.^28^	Acute myocardial infarction (1 patient)				
Kano et al.^31^	Effects of radiosurgery (10 patients), approximately 7 months after treatment				
Lee et al.^40^	Effects of radiosurgery (11 patients), approximately 6 months after treatment				
Lv et al.^41^	Seizure control: excellent (21 patients), good (4 patients), fair (2 patients), and poor (3 patients)				
Wikholm et al.^49^	Impaired memory (8 patients), with a later improvement (4 patients)				
Zabel-du Bois et al.^26^	15/50 had visual deterioration or visual field changes before radiosurgery. Deficits were maintained in 87% of the cases, 1 patient resolved completely and 1 improved partially. Asymptomatic focal edema developed in 42% of patients 7.5 months after radiosurgery				
Shekhtman et al.^23^	Glasgow Outcome Scale score: 1 (2 patients), 2 (0), 3 (1 patient), 4 (12 patients) and 5 (15 patients)				

N/S = not specified; C = cases; CT = controls.

* Hemiparesis (15 cases), speech disorder (11 cases, including 4 aphasia, 3 dysarthria, and 4 unspecified cases), visual disturbance (10 cases, including 5 hemianopia, 2 diplopia, and 3 unspecified cases), ataxia (2 cases), paresthesia (2 cases), facial paralysis (2 cases), permanent weakness in the right hand (1 case), and unilateral deafness (1 case).

Twenty-three studies provided information on long-term obliteration/cure rates. Notably, 552 complete obliterations were reported in 1,071 patients (51.4%), with a mean duration of 36 months. Additionally, nine studies reported 36 long-term deaths in a population of 889 patients (4%). Of these, three studies reported that deaths occurred due to hemorrhagic events. The shortest follow-up time until death was 30 days, and the longest was 56.04 months (**Table 3**).

Data on long-term recanalization of AVMs were only described in five studies. A total of 231 patients were followed up, of whom eight cases of recanalization (3.5%) were identified. Of these, one was identified after 1 year of follow-up, whereas four were identified at an interval of 2.5–4 months. Additionally, other outcomes that did not fit our study variables, such as the long-term effects of radiosurgery, acute myocardial infarction, and memory impairment (eight patients), were also reported (**Table 3**).

## DISCUSSION

Our study showed that AVMs generally affect young adults (30–40 years old) and are more prevalent in males. The most common clinical presentation was hemorrhage, followed by headaches, seizures, and focal neurological deficits. The patients selected for endovascular treatment, either alone or in combination, mostly had Spetzler–Martin grade II or III AVMs. The most frequent long-term complications were hemorrhagic events (8.7%) and neurological deficits (6.3%). In most studies, the follow-up time until the onset of these complications was >2 years. Notably, 51.4% of patients (552 of 1,071) achieved complete AVM obliteration after an average of 36 months of follow-up, whereas recanalization was identified in 3.5% patients (8 of 231). Finally, 4% of patients (36 of 889) died, mostly due to hemorrhagic events.

### Long-term hemorrhagic events

The risk of long-term bleeding varies according to treatment type. Procedures that have a direct effect on AVMs, such as microsurgery and embolization, have a relatively short post-treatment bleeding latency period, varying from days to months. However, in radiosurgery, the latency period between the end of the procedure and hemorrhage can vary from 2 to 3 years.^12,23^ The clinical presentation (with or without initial bleeding) also affects the risk of long-term bleeding. The risk of new hemorrhage in the first year is 6.5–32.9% in patients with a clinical presentation of hemorrhagic events (ruptured AVMs), whereas a lower risk of 0–3.6% is observed for patients without hemorrhage in the initial clinical presentation.^23,21,24,25^

Lv et al.^21^ analyzed the bleeding rates in patients with AVMs after embolization alone or in combination and reported 20 long-term bleeding events, 11 of which involved hemorrhage at initial clinical presentation. Further, the mean follow-up time until the appearance of a bleeding event in the groups without and with bleeding at the initial presentation was 3.5 and 7.3 years, respectively.

Zabel-du Bois et al.^26^ identified six hemorrhagic events in a group of 50 patients who underwent embolization and radiosurgery, with an average time of 8.5 months. Further, they reported the lowest rate of obliteration after embolization in patients with hemorrhagic events.

### Long-term neurological deficits

The literature indicates a 4–18% risk of permanent neurological deficit, which may vary according to the embolizing material, technique used, and AVM characteristics.^27^ Hartmann et al.^28^ reported a 14% permanent neurological deficit rate in a series of 233 cases and a total of 545 embolizations.

Pierot et al.,^22^ in their retrospective cohort study, analyzed 20 patients who underwent 77 embolizations followed by radiosurgery, with Onyx as the embolizing material. Worsening of the neurological clinical presentation was reported in only one patient, with a pre-mRs score of 1 evolving to 2, and the overall neurological clinical results were classified as satisfactory.

Starke et al.^29^ identified 19 patients with post-embolization neurological deficits (9 with a mRs score ≤ 2 and 10 with a mRs score > 2) among 202 patients who underwent embolization. After a mean follow-up of 43.4 months, five persistent neurological deficits were identified, which were equivalent to 1% of the procedures and 2% of the patients. The authors concluded that most neurological deficits were transient and that even moderate and severe deficits were likely to improve over time.

### Complete obliteration and death

Recently, the role of endovascular therapy in the treatment of AVMs has improved. However, low cure rates (16–32.8%) have been reported using endovascular embolization as a single treatment modality, with mortality rates ranging from 0% to 7.5%. More recently, some studies reported an obliteration rate of ≥ 51% using Onyx, indicating advancement in the role of endovascular therapy in the treatment of AVMs.^30,28,23^ Iosif et al.^17^ reported a high cure rate (95%) in 73 patients with low-grade AVMs using Onyx as an embolizing agent within an angiographic follow-up time of 12–18 months, indicating the superiority of therapeutic embolization to small-sized AVMs. However, randomized clinical trials are required to confirm the superiority of this therapeutic approach.

The factors that determine the success of endovascular therapy depend on the selection of AVM characteristics. Abud et al.,^16^ in a series of 17 patients undergoing embolization to cure AVMs using Onyx as an embolizing agent, concluded that the success of embolization was dependent on small AVM size (1–3 cm), location other than in the brainstem or deep brain structures, with renal arteries easily accessible by a microcatheter with the possibility of reflux of 2–3 cm, and a clear location of the proximal parts of the drainage veins such that the operator recognizes the venous filling with Onyx in time.

The role of embolization in radiosurgery varies from occlusion of aneurysms to decreasing the volume of AVMs, decreasing the radiation doses to which the nidus needs to be exposed, and decreasing the amount of cerebral blood flow. However, the obliteration is slow to achieve. Kano et al.^31^ described cure rates of 35%, 53%, 55%, and 59% at 3, 4, 5, and 10 years, respectively. Singfer et al.^32^ reported a 77.2% cure rate after an average of 5 years of follow-up.

The cure rate also varies according to the embolization agent used. Liu et al.^33^ evaluated the results of AVM embolization using Glubran 2 as an embolizing agent. Of the 31 patients who underwent embolization, 27 had an obliteration rate of 100%, whereas 4 had an obliteration rate of 90%–99%. The duration of clinical and angiographic follow-ups ranged from 3 to 6 months.

### Recanalization of AVMs

Recanalization (or recurrence) of AVMs is defined as new radiological evidence or a new event, commonly hemorrhage, in a patient who underwent angiographic examination after treatment, indicating complete obliteration of the AVM nidus. The use of new treatment techniques has enabled lower recanalization rates and, consequently, less AVM recurrence.^34^

Panagiatopoulos et al.^35^ reported complete obliteration in 20 patients and four AVM recurrences 2.5–4 months after embolization through angiographic examination among 80 patients (average of 1.45 embolizations per patient), using Onyx as an embolizing agent. Of the four recurrences, two were identified as minimal.

### Other complications and long-term outcomes

Kano et al.^31^ reported adverse effects related to radiosurgery, which were unrelated to previous embolization, in an average follow-up period of 7 months in 10 patients (8.3%). One patient required a surgical procedure because of cyst formation and cerebral edema 8 months after radiosurgery. Postoperatively, the same patient developed persistent aphasia and hemiparesis.

Valavanis et al.,^36^ in their study including 1,114 patients, reported the development of amyotrophic lateral sclerosis (ALS) in seven patients after an average follow-up of 132 months. Notably, the low production of vascular endothelial growth factors by AVMs with significant angiogenesis, possibly due to several embolization procedures, may contribute to the development of ALS.

Shekhtman et al.,^23^ in their case-control study, compared the outcomes of patients who underwent embolization and microsurgery (n = 40) with those who underwent microsurgery alone (n = 40). Long-term outcomes were assessed in 30 patients who underwent embolization and microsurgery using the Glasgow Outcome Score (GOS), with an average follow-up time of 27.6 months. Of these, 2, 0, 1, 12, and 15 patients had GOS 1, 2, 3, 4, and 5, respectively. Of the 40 patients that underwent microsurgery only, 1, 2, 0, 19, and 18 had GOS 1, 2, 4, and 5, respectively.

### Study limitations

All studies included in this review were cohort or case-control studies with inherent limitations of observational studies, such as selection bias, sample size, and losses during follow-up. Most of the studies included in this review were hospital-based studies in single centers, increasing the risk of bias and limiting the representativeness of the data. In addition, the generalizability of the results was limited owing to the limitations in the selection of cases and controls. Notably, only a few studies selected for this review had a control group, and the follow-up period was small.

Although most of the included studies used current techniques, studies conducted in the 1990s and early 2000s were limited by the lack of technological advances in endovascular therapy, introduction of catheters, and development of new embolic agents. Finally, the outcomes may be influenced by the heterogeneity of the studies in terms of evaluation methods and follow-up protocols. Consequently, multicenter and randomized studies involving a larger sample size, with sufficiently long follow-up periods are crucial to overcome these limitations.

## CONCLUSIONS

Embolization of AVMs is a therapeutic strategy exhibiting greater safety in microsurgical or radiosurgical treatment, with high cure rates depending on the characteristics of the AVMs, technique, and resources used during the endovascular procedure. Further, long-term complications are low, and outcomes are considered positive, especially in patients who undergo combination therapies.

## References

[1] Finby N, Begg CF (1965). Arteriovenous malformation of the brain.. N Y State J Med..

[2] Mast H, Young WL, Koennecke HC (1997). Risk of spontaneous haemorrhage after diagnosis of cerebral arteriovenous malformation.. Lancet..

[3] Laakso A, Hernesniemi J (2012). Arteriovenous malformations: epidemiology and clinical presentation.. Neurosurg Clin N Am..

[4] Galaktionov DM, Dubovoy AV, Kiselev VS (2017). Kombinirovannoe lechenie arteriovenoznykh mal’formatsiĭ golovnogo mozga s ispol’zovaniem éndovaskuliarnogo i mikrokhirurgicheskogo metodov [Combination treatment of cerebral arteriovenous malformations using endovascular and microsurgical techniques].. Zh Vopr Neirokhir Im N N Burdenko..

[5] van Beijnum J, van der Worp HB, Buis DR (2011). Treatment of brain arteriovenous malformations: a systematic review and meta-analysis.. JAMA..

[6] Zuurbier SM, Al-Shahi Salman R (2019). Interventions for treating brain arteriovenous malformations in adults.. Cochrane Database Syst Rev..

[7] Mohr JP, Parides MK, Stapf C (2014). Medical management with or without interventional therapy for unruptured brain arteriovenous malformations (ARUBA): a multicentre, non-blinded, randomised trial.. Lancet..

[8] Ogilvy CS, Stieg PE, Awad I (2001). Recommendations for the management of intracranial arteriovenous malformations: a statement for healthcare professionals from a special writing group of the Stroke Council, American Stroke Association.. Circulation..

[9] Bendok BR, El Tecle NE, El Ahmadieh TY (2014). Advances and innovations in brain arteriovenous malformation surgery.. Neurosurgery..

[10] Wells G, Shea B, O’Connell D (2013). The Newcastle-Ottawa Scale (NOS) for assessing the quality of nonrandomized studies in meta-analyses..

[11] Yang W, Wei Z, Wang JY (2016). Long-term Outcomes of Patients With Giant Intracranial Arteriovenous Malformations.. Neurosurgery..

[12] Yang W, Hung AL, Caplan JM (2016). Delayed Hemorrhage After Treatment of Brain Arteriovenous Malformations (AVMs).. World Neurosurg..

[13] Dehdashti AR, Thines L, Willinsky RA (2010). Multidisciplinary care of occipital arteriovenous malformations: effect on nonhemorrhagic headache, vision, and outcome in a series of 135 patients.. J Neurosurg..

[14] Singfer U, Hemelsoet D, Vanlangenhove P (2017). Unruptured Brain Arteriovenous Malformations: Primary ONYX Embolization in ARUBA (A Randomized Trial of Unruptured Brain Arteriovenous Malformations)-Eligible Patients.. Stroke..

[15] Xu F, Ni W, Liao Y (2011). Onyx embolization for the treatment of brain arteriovenous malformations.. Acta Neurochir (Wien)..

[16] Abud DG, Riva R, Nakiri GS (2011). Treatment of brain arteriovenous malformations by double arterial catheterization with simultaneous injection of Onyx: retrospective series of 17 patients.. AJNR Am J Neuroradiol..

[17] Iosif C, de Lucena AF, Abreu-Mattos LG (2019). Curative endovascular treatment for low-grade Spetzler-Martin brain arteriovenous malformations: a single-center prospective study.. J Neurointerv Surg..

[18] Mendes GA, Iosif C, Silveira EP (2016). Transvenous Embolization in Pediatric Plexiform Arteriovenous Malformations.. Neurosurgery..

[19] Weber W, Kis B, Siekmann R (2007). Preoperative embolization of intracranial arteriovenous malformations with Onyx.. Neurosurgery..

[20] Cronqvist M, Wirestam R, Ramgren B (2006). Endovascular treatment of intracerebral arteriovenous malformations: procedural safety, complications, and results evaluated by MR imaging, including diffusion and perfusion imaging.. AJNR Am J Neuroradiol..

[21] Lv X, Wu Z, Jiang C (2010). Endovascular treatment accounts for a change in brain arteriovenous malformation natural history risk.. Interv Neuroradiol..

[22] Pierot L, Kadziolka K, Litré F, Rousseaux P (2013). Combined treatment of brain AVMs with use of Onyx embolization followed by radiosurgery.. AJNR Am J Neuroradiol..

[23] Shekhtman OD, Maryashev SA, Eliava SS (2015). Combined treatment of cerebral arteriovenous malformations. Experience of the Burdenko Neurosurgical Institute.. Zh Vopr Neirokhir Im N N Burdenko..

[24] da Costa L, Wallace MC, Ter Brugge KG (2009). The natural history and predictive features of hemorrhage from brain arteriovenous malformations.. Stroke..

[25] Halim AX, Johnston SC, Singh V (2004). Longitudinal risk of intracranial hemorrhage in patients with arteriovenous malformation of the brain within a defined population.. Stroke..

[26] Zabel-du Bois A, Milker-Zabel S, Huber P, Schlegel W, Debus J (2007). Risk of hemorrhage and obliteration rates of LINAC-based radiosurgery for cerebral arteriovenous malformations treated after prior partial embolization.. Int J Radiat Oncol Biol Phys..

[27] Choi JH, Mohr JP (2005). Brain arteriovenous malformations in adults.. Lancet Neurol..

[28] Hartmann A, Pile-Spellman J, Stapf C (2002). Risk of endovascular treatment of brain arteriovenous malformations.. Stroke..

[29] Starke RM, Komotar RJ, Otten ML (2009). Adjuvant embolization with N-butyl cyanoacrylate in the treatment of cerebral arteriovenous malformations: outcomes, complications, and predictors of neurologic deficits.. Stroke..

[30] Blauwblomme T, Bourgeois M, Meyer P (2014). Long-term outcome of 106 consecutive pediatric ruptured brain arteriovenous malformations after combined treatment.. Stroke..

[31] Kano H, Kondziolka D, Flickinger JC (2012). Stereotactic radiosurgery for arteriovenous malformations after embolization: a case-control study.. J Neurosurg..

[32] Singfer U, Hemelsoet D, Vanlangenhove P (2017). Unruptured Brain Arteriovenous Malformations: Primary ONYX Embolization in ARUBA (A Randomized Trial of Unruptured Brain Arteriovenous Malformations)-Eligible Patients.. Stroke..

[33] Liu J, Lv M, Lv X (2014). Curative glubran 2 embolization of cerebral arteriovenous malformations patient selection and initial results.. Interv Neuroradiol..

[34] Aboukaïs R, Vinchon M, Quidet M (2017). Reappearance of arteriovenous malformations after complete resection of ruptured arteriovenous malformations: true recurrence or false-negative early postoperative imaging result?. J Neurosurg..

[35] Panagiotopoulos V, Gizewski E, Asgari S (2009). Embolization of intracranial arteriovenous malformations with ethylene-vinyl alcohol copolymer (Onyx).. AJNR Am J Neuroradiol..

[36] Valavanis A, Schwarz U, Baumann CR, Weller M, Linnebank M (2014). Amyotrophic lateral sclerosis after embolization of cerebral arterioveneous malformations.. J Neurol..

[37] Al-Smadi AS, Ansari SA, Shokuhfar T (2019). Safety and outcome of combined endovascular and surgical management of low grade cerebral arteriovenous malformations in children compared to surgery alone.. Eur J Radiol..

[38] Consoli A, Scarpini G, Rosi A (2014). Endovascular treatment of unruptured and ruptured brain arteriovenous malformations with Onyx18: a monocentric series of 84 patients.. J Neurointerv Surg..

[39] de Castro-Afonso LH, Nakiri GS, Oliveira RS (2016). Curative embolization of pediatric intracranial arteriovenous malformations using Onyx: the role of new embolization techniques on patient outcomes.. Neuroradiology..

[40] Lee CC, Chen CJ, Ball B (2015). Stereotactic radiosurgery for arteriovenous malformations after Onyx embolization: a case-control study.. J Neurosurg..

[41] Lv X, Li Y, Jiiang C, Yang X, Wu Z (2010). Brain arteriovenous malformations and endovascular treatment: effect on seizures.. Interv Neuroradiol..

[42] Mathis JA, Barr JD, Horton JA (1995). The efficacy of particulate embolization combined with stereotactic radiosurgery for treatment of large arteriovenous malformations of the brain.. AJNR Am J Neuroradiol..

[43] Pérez-Higueras A, López RR, Tapia DQ (2005). Endovascular Treatment of Cerebral AVM: Our Experience with Onyx.. Interv Neuroradiol..

[44] Pierot L, Cognard C, Herbreteau D (2013). Endovascular treatment of brain arteriovenous malformations using a liquid embolic agent: results of a prospective, multicentre study (BRAVO).. Eur Radiol..

[45] Raupp EF, Fernandes J (2005). Does treatment with N-butyl cyanoacrylate embolization protect against hemorrhage in cerebral arteriovenous malformations?. Arq Neuropsiquiatr..

[46] Sahlein DH, Mora P, Becske T, Nelson PK (2012). Nidal embolization of brain arteriovenous malformations: rates of cure, partial embolization, and clinical outcome.. J Neurosurg..

[47] Umansky D, Corn BW, Strauss I (2018). Combined treatment approach to cerebral arteriovenous malformation in pediatric patients: stereotactic radiosurgery to partially Onyx-embolized AVM.. Childs Nerv Syst..

[48] van Rooij WJ, Jacobs S, Sluzewski M (2012). Curative embolization of brain arteriovenous malformations with onyx: patient selection, embolization technique, and results.. AJNR Am J Neuroradiol..

[49] Wikholm G, Lundqvist C, Svendsen P (2001). The Göteborg cohort of embolized cerebral arteriovenous malformations: a 6-year follow-up.. Neurosurgery..

[50] Jordan J, Llibre JC, Vazquez F (2014). Predictors of neurological deficit after endovascular treatment of cerebral arteriovenous malformations and functional repercussions in prospective follow-up.. Neuroradiol J..

